# Editorial: Genetic analysis of reproductive traits in livestock

**DOI:** 10.3389/fgene.2022.1116038

**Published:** 2023-01-04

**Authors:** Aixin Liang, Yang Zhou, Hasan Riaz, John S. Davis

**Affiliations:** ^1^ College of Animal Science and Technology, Huazhong Agricultural University, Wuhan, China; ^2^ VA Nebraska-Western Iowa Health Care System, Department of biosciences, COMSATS University Islamabad, Sahiwal, Pakistan; ^3^ Department of Obstetrics and Gynecology, University of Nebraska Medical Center, Omaha, NE, United States

**Keywords:** reproduction, SNP, GWAS, RNA-seq, molecular pathway

Reproduction is one of the most important traits of livestock to maintain the continuity of the species. Improving the reproductive performance of livestock is of importance and tightly related to the selection intensity and production costs (Schmidt et al., 2019). Candidate gene and high throughput studies have been used to better understand the genetic basis of reproductive traits over the last decades (Óvilo and Valdovinos, 2012). However, the identification and analysis of specific functional and positional variants and molecular regulatory pathways influencing reproductive efficiency remain a challenging task. Therefore, the goal of this Research Topic is to present current knowledge about genetic factors affecting reproduction in animals and present state-of-the-art methods for studying genetic influences on reproductive phenotypes. Based on female or male reproductive traits, the thirteen articles published in this Research Topic cover many different species such as pig, cattle, sheep, rabbit, chicken, duck, and turkey. This article collection shows recent advances, recent technologies, and challenges in livestock reproduction.

High-throughput transcriptome sequencing (RNA-Seq) has become the main approach to identify the key genes related to reproductive traits. The work of Mao et al. describe the use of RNA-seq to screen key genes and lncRNAs that affect the fecundity of pigs. The report highlights an important regulatory role that lncRNA MSTRG.3902.1 may play in rpFSH-induced ovulation by affecting the target gene NR5A2 (nuclear receptor subfamily 5, group A, member 2). Likewise, Du et al. use RNA-seq technology to identify differentially expressed genes in ewe adrenal glands under different photoperiod treatments, and identify several novel mRNA, miRNAs, and lncRNAs, which may regulate sheep seasonal estrus. Using RNA-seq technology, Zhang et al. identified several mRNAs (e.g., *GAMT*, *SOHLH1*, *DMC1*, *MACROD1*, *WNT2B*, *SPIN1*, *CRH*, *TTR*, and *WISP1*) that may have direct or indirect functions in the initiation of puberty, which may provide new insight into the mechanisms that initiate puberty in sheep. Moreover, Ross et al. provided a case study that combined information from multiple expression datasets such as RNA-seq, ISO-seq and CAGE-seq, and identified several genes relevant to fertility in Brahman cattle. They demonstrated tissue-specific expression of the selected genes, allele-specific expression, variation in transcription start sites, and untranslated regions. The integration of RNA-seq and other sequencing technologies will be a viable alternative to effectively improve the accuracy of candidate gene selection.

In addition to RNA-seq technology, genome-wide association studies (GWASs) and whole-genome sequencing are also widely used to identify key single nucleotide polymorphisms (SNPs) and candidate genes that correspond to reproductive traits. By using GWASs, Mo et al. identified a total of 29 candidate SNPs for seven litter-size traits and four teat-number traits in Bama Xiang pigs. By using GWASs and haplotype-sharing analysis, Xu et al. observed candidate genes and haplotypes that were significantly associated with egg production traits in laying ducks. In addition, Zhang et al. reported on 10 important candidate genes related to bone traits, and two bone-related pathways such as osteoclast differentiation and MAPK (Mitogen Activated Protein Kinase) signaling pathway in laying chicken populations using whole-genome pooled sequencing. By integrated analysis of GWASs and transcriptome data, the study of Shi et al. identified 7 significant SNPs and proposed 28 candidate genes associated with sow milk production, 10 of which were key candidates. Compared to the traditional the cumulative model, Makanjuola et al. demonstrated that random regression models using pedigree and genomic information can achieve a higher predictive ability for analyzing longitudinal traits such as fertile eggs set in the incubator (FERT), hatch of fertile (HOF), and hatch of set (HOS) in turkeys.

It has been reported that splicing isoforms may exert distinct functions in reproductive physiological processes, such as progesterone receptor isoforms (Rekawiecki et al., 2011) and prolactin receptor isoforms (Binart et al., 2010). The article of Kern et al. describe four isoforms of preferentially expressed antigen in melanoma Y-linked (PRAMEY) in the bovine testis and spermatozoa. The study implicates that the 58 and 30 kDa PRAMEY isoforms are involved in spermatogenesis, whereas the 13 kDa PRAMEY isoform is responsible for sperm maturation and sperm motility.

Microbial communities in the reproductive tract are involved in the maintenance of host fertility and health (Feng and Liu, 2022). Endometrial inflammation is common in postpartum dairy cows, and alterations in the uterine microbiota are associated with perinatal disease. The study of Kudo et al. revealed that *Trueperella* is present in higher abundance in the uterus and vagina of the endometritis group and is negatively correlated to the abundance of *Lactobacillus.* As mentioned in their article, their findings are helpful for predicting endometritis and developing prevention or treatment strategies.

Myostatin (MSTN) is regarded as a negative regulator of muscle development and regeneration, and natural mutations of *MSTN* result in an obvious double muscle phenotypic effect in cattle, dogs, sheep, and pigs. In this Research Topic, Zheng et al. developed a heritable double muscle buttocks in rabbits *via MSTN* mutation using the CRISPR/Cas9 system. This may improve rabbit meat production efficiency and promote the development of the rabbit industry.

In summary, integration of the available technological approaches provides more powerful tools for the identification of novel functional candidate genes, specific genetic variants, and molecular pathways affecting reproductive traits. The CRISPR/Cas9 system is an efficient genome editing tool for the validation of functional genes in relation to reproduction, which may significantly improve reproductive efficiency in livestock.
